# Supplementary material for: Thermal transport mechanisms in ZIFs
Source: Nat Commun. 2025 Nov 27;16:11682. doi: 10.1038/s41467-025-66510-4 (PMC12749508; doi:10.1038/s41467-025-66510-4)
Supplement: Supplementary file 1 — Supplementary Information [file 41467_2025_66510_MOESM1_ESM.pdf]

Supplementary Information: Thermal Transport Mechanisms in ZIFs

Xiaoqi Zhang [1], Senja Barthel [2], Yutao Li [1], Berend Smit *[1], and Raffaela Cabriolu †[3]

[1]Institut des Sciences et Ingénierie Chimiques, École Polytechnique Fédérale de Lausanne (EPFL), Rue de l'Industrie 17, Sion, 1951, Switzerland

[2]Department of Mathematics, Vrije Universiteit Amsterdam, De Boelelaan 1111, Amsterdam, 1081 HV, Netherlands

[3]Department of Physics, Norwegian University of Science and Technology (NTNU), Høgskoleringen 5, Realfagbygget D5-149, Trondheim, 7491, Norway

*berend.smit@epfl.ch

†raffaela.cabriolu@ntnu.no

Contents

1 Correlation with void fraction 3

2 Automatic thermal conductivity fitting 4

3 Bulk modulus of ZIFs 7

4 Effect of linker mass on thermal conductivity 8

5 Decomposition of the thermal conductivity 9

6 ZIFs with SOD and BCT net topologies 12

7 Thermal circuit conductivity fitting 13

8 Deviation of the thermal circuit prediction 16

9 Remarks on the circuit model 17

1 Correlation with void fraction

Figure S1 shows the thermal conductivity as a function of the void fraction. The void fraction of a MOF was computed using Zeo++[1] (version 0.3) with helium as the probe molecule, assuming a radius of 1.3 Å. In general, void fraction and thermal conductivity exhibit a negative correlation. However, as discussed in the main text, no universal trend applies to all four organic linkers.

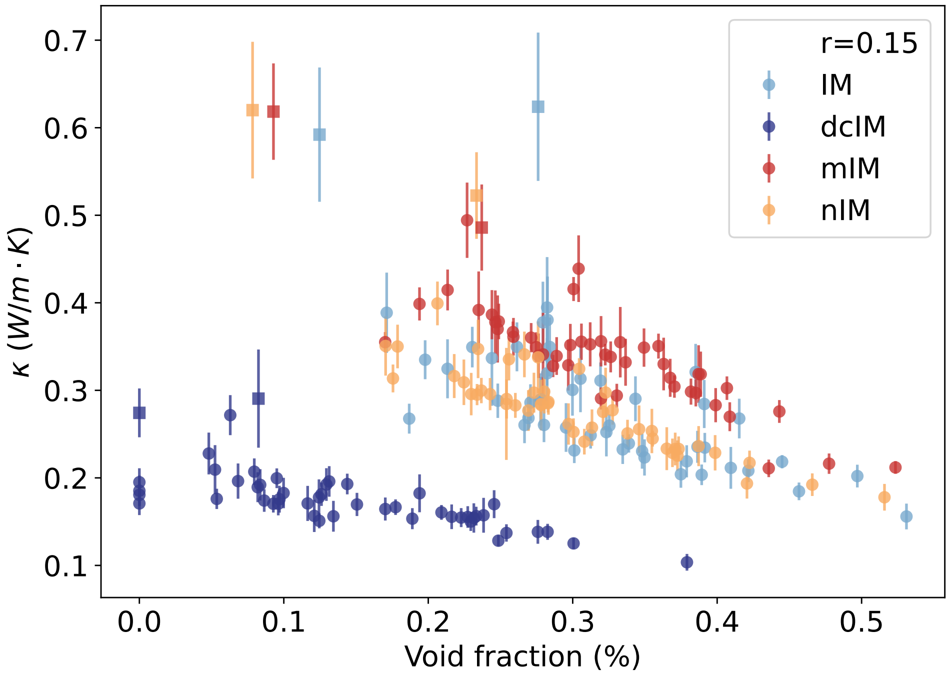

Supplementary Figure S1: Correlation between thermal conductivity and void fraction. A negative linear correlation between void fraction and thermal conductivity is observed for each specific combination of metal nodes and organic ligands.

2 Automatic thermal conductivity fitting

The thermal conductivity of anisotropic materials is characterized by a thermal conductivity tensor, expressed as:

$$\kappa = \begin{bmatrix} \kappa_{xx} & \kappa_{xy} & \kappa_{xz} \\ \kappa_{yx} & \kappa_{yy} & \kappa_{yz} \\ \kappa_{zx} & \kappa_{zy} & \kappa_{zz} \end{bmatrix} \tag{S1}$$

Each coefficient is computed using a Green-Kubo relation involving the heat flux. This calculation involves an integration over time using the autocorrelation of the heat flux. A practical difficulty is that the longer the correlation time, the smaller the number of samples, and the integration is dominated by the noise. Hence, we need to ensure that our simulation is sufficiently long that the integral can be safely truncated before the noise dominates the integration. If we observe a plateau in the estimated thermal conductivity coefficient as a function of the truncation of the integration time, we can use the plateau value as our estimate.

An example of thermal conductivity versus correlation time for a specific structure (ZIF(nIM) with IFY net topology) is shown in Fig. S2a, with a total simulation time of approximately 50 ns. The structures in our database are mostly orthogonal with slight distortions. As a result, the off-diagonal terms $(\kappa_{xy}, \kappa_{xz}, \kappa_{yz})$ fluctuate around zero, as illustrated in Fig. S2a. Thus, we focus on the diagonal elements $(\kappa_{xx}, \kappa_{yy}, \kappa_{zz})$ and their average, as they effectively represent the structure's thermal conductivity. We divided the total simulation into segments of 10 ns and 5 ns (Figs. S2b to S2d for $x-$,$y-$, and $z-$directions, respectively) to estimate the average and standard deviation of the thermal conductivity from independent trajectories.

We developed an algorithm to identify the correlation time window in which the thermal conductivity values reach a stable plateau. Five correlation time windows were selected, each with a fixed duration of 10 ps, centered at 10 ps, 20 ps, 30 ps, 40 ps, and 50 ps. Within each window, we performed a first-order polynomial fit to the entire trajectory, as illustrated by the line segments in Figs. S2e to S2g. The window with the lowest absolute slope was selected as the most stable region. We then computed the average

thermal conductivity within this optimal window for the full trajectory, as well as for the 10 ns and 5 ns segments, shown in Figs. S2h to S2j and Figs. S2k to S2m, respectively. The error bars for thermal conductivity were determined from the standard deviations of these segment averages.

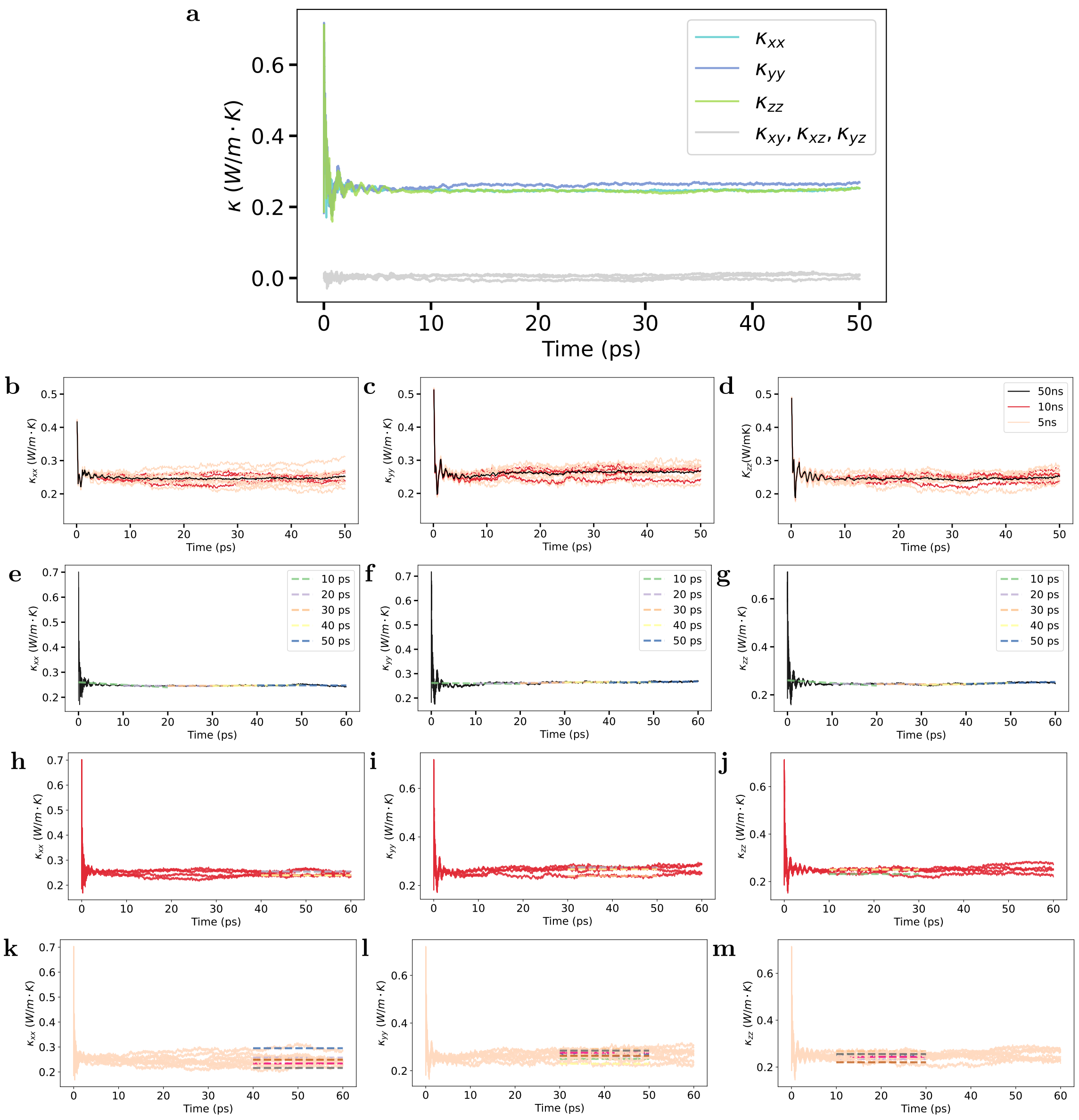

Supplementary Figure S2: Thermal conductivity as a function of correlation time for ZIF(nIM) with IFY net topology. (a) Diagonal and off-diagonal components of the thermal conductivity over the entire trajectory. (b)—(d) Thermal conductivity obtained from trajectories of 50 ns, 10 ns, and 5 ns for the x-, y-, and z-directions, respectively. (e)—(g) Fitting procedure using the 50 ns trajectory for the x-, y-, and z-directions, respectively. (h)—(j) Average thermal conductivity computed for each 10 ns segment in the x-, y-, and z-directions, respectively. (k)—(m) Average thermal conductivity computed for each 5 ns segment in the x-, y-, and z-directions, respectively.

3 Bulk modulus of ZIFs

Figure S3 is plotted using the data from previous work[2]. This figure shows that ZIFs(mIM) and ZIFs(nIM) generally exhibit a higher bulk modulus compared to ZIFs(IM), while ZIFs(dcIM) fall within a similar range. The trend aligns with Fig. 4 in the main text, which indicates the relationship between mechanical stability and thermal conductivity.

In this figure, we also highlight the bulk modulus of ZIFs with the SOD and BCT net topologies, which have a relatively large bulk modulus.

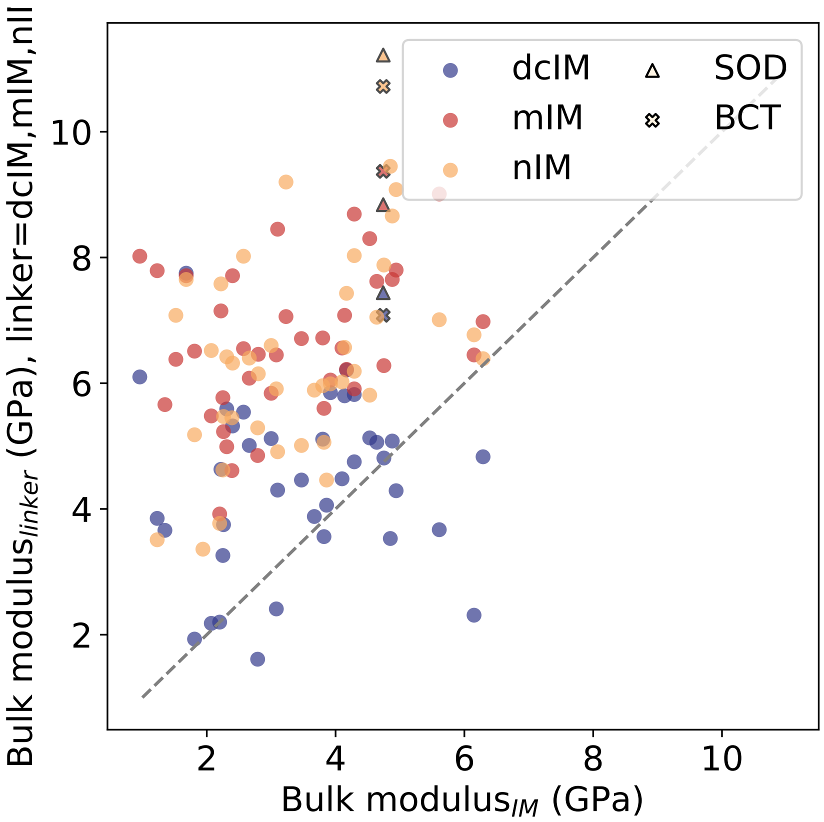

Supplementary Figure S3: Bulk modulus of ZIFs(dcIM), ZIFs(mIM), and ZIFs(nIM) against that of ZIFs(IM). ZIFs with two special net topologies — SOD and BCT, are highlighted by different markers.

4 Effect of linker mass on thermal conductivity

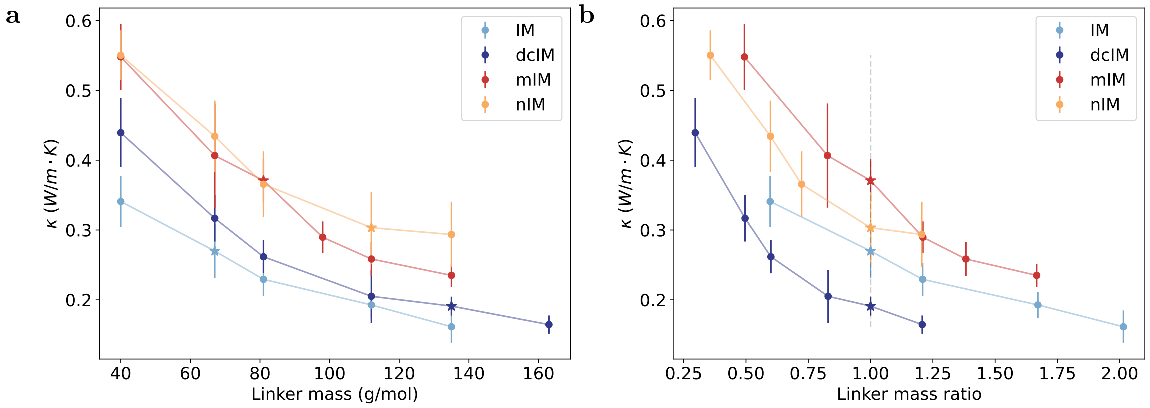

Supplementary Figure S4: Thermal conductivity change against (a) the ligands' masses and (b) the ratio of modified ligand's mass and the original mass of different ZIFs with the MEP net topology. The original mass is indicated with a star.

The mass of the linkers has a significant effect on the overall thermal conductivity. To gain insights into how it impacts thermal conductivity, we carried out simulations in which we (artificially) changed the mass of the ligands.

Figures S4a and S4b show the change of thermal conductivity when we scale the masses of the four linkers such that they all have the same total mass. For each of the linkers, the results show the expected behavior; increasing the mass of the linker decreases the thermal conductivity. In addition, if the masses of the linkers were equal, we would observe the thermal conductivity: κ(ZIF(MEP,nIM)) $>$ κ(ZIF(MEP,mIM)) $>$ κ(ZIF(MEP,dcIM)) $>$ κ(ZIF(MEP,IM)) —— independent of the absolute linker mass. Figure S4b presents the same data, but with linker mass scaled by its actual mass for each linker, shifting the observed order accordingly.

Hence, if we add functional groups to the linker, we make the linker heavier, which will decrease the thermal conductivity. However, this is not the only effect, as is seen in Figs. S4a and S4b; if we eliminate the effect of increasing the mass, we see, in fact, an increase in the thermal conductivity that depends on the functional group.

5 Decomposition of the thermal conductivity

LAMMPS with many-body interactions correction version computes the instantaneous heat flux as[3]:

$$\mathbf{J}_{\text{heat}}V = \sum_i E_i \mathbf{v}_i + \sum_{m=2}^{4} \sum_{\phi \in \mathscr{P}_m} \left(\frac{1}{m} \sum_{[i,j] \in [\phi]^2} [\mathbf{r}_{ij} (\mathbf{F}_{i\phi} \cdot \mathbf{v}_i - \mathbf{F}_{j\phi} \cdot \mathbf{v}_j)] \right), \qquad \text{(S2)}$$

where $\mathscr{P}_m$ is $m-$body potentials defined by the UFF4MOF force field[4, 5], which includes bonded interactions and van der Waals (vdW) interactions. The first term, $\sum_i E_i \mathbf{v}_i$, corresponds to the convective heat flux arising from atomic motion within the system. However, this contribution should be negligible, as the center of mass of the system is expected to remain stationary. E_i is the total energy associated with atom i moving with velocities $\mathbf{v}_i$. The second term accounts for the potential (or virial) contribution to the heat flux. It is expressed as a sum over all many-body potential terms of different orders m, from two-body to four-body interactions, each denoted by $\phi \in \mathscr{P}m$. Within each potential term ϕ, all pairs of atoms i and j that participate in that interaction are considered. The vector $\mathbf{r}_{ij} = \mathbf{r}_i - \mathbf{r}_j$ denotes the displacement between atoms i and j, while $\mathbf{F}_{i\phi}$ is the component of the force acting on atom i due to the potential term ϕ. The inner product $(\mathbf{F}_{i\phi} \cdot \mathbf{v}_i - \mathbf{F}_{j\phi} \cdot \mathbf{v}_j)$ represents the rate of energy transfer between atoms i and j, and the prefactor $1/m$ ensures that the contribution of each m-body potential is properly normalized. Together, these two terms describe how energy is transported at the microscopic level, both through atomic motion and through interatomic interactions.

By decomposing the heat flux into individual components and correlating them with the total heat flux, the corresponding contributions to the thermal conductivity can be determined. [6] We applied this decomposition to ZIFs with six different net topologies to further investigate the effect of functional groups on thermal conductivity.

Unlike the calculation of total thermal conductivity mentioned in Section 4, we performed the same equilibration procedure but a shorter production simulation with four different velocity initialization seeds. Following each equilibration, a 500 ps NVE simulation was performed to record the heat flux components for computational efficiency.

By computing the average contributions of these four trajectories, we make sure that the results are converged. The fitting windows for locating the thermal conductivity plateau are adjusted accordingly. The thermal conductivity was then computed as the average across these four trajectories. Consequently, while the values presented in Fig. S5 differ from those in other figures, they remain within the error bars.

We obtained a general trend for the thermal conductivity κ:

$$\kappa(\text{ZIFs(mIM)}) > \kappa(\text{ZIFs(nIM)}) \approx \kappa(\text{ZIFs(IM)}) > \kappa(\text{ZIFs(dcIM)}).$$

In Fig. S5, we show the decomposition of the contributions to the thermal conductivity for some net topologies that follow this trend. If we add functional groups, there are two contributions: we change the mass of the linker (see Section 4) and the mechanical stability (see Section 3). In addition, increased vdW interactions can generate a secondary network.[2]

Figure S5 shows that the vdW contributions (yellow bars) to the thermal conductivity of ZIFs with functional groups ZIFs(dcIM), ZIFs(mIM), and ZIFs(nIM) are higher than those of ZIFs(IM). This suggests that functional groups introduce secondary heat conduction pathways and increase the mechanical stability of the network. While these effects enhance heat conduction, the increased mass of functional groups counteracts this by reducing thermal conductivity.

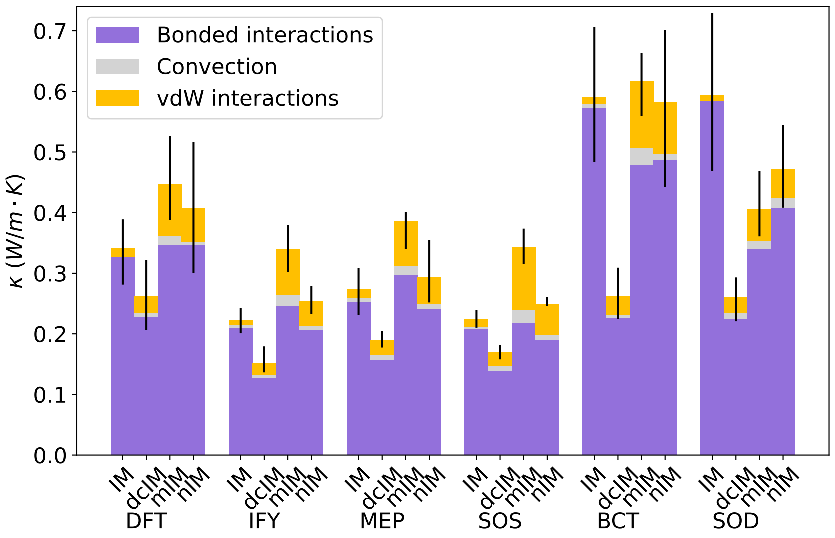

Supplementary Figure S5: Decomposition of thermal conductivity into three contributions: convection, bonded interactions, and vdW interactions. Introducing functional groups in the structures increases the ligand's mass and vdW contributions to the thermal conductivity.

6 ZIFs with SOD and BCT net topologies

Structures with net topology BCT and SOD exhibit exceptionally high thermal conductivity in Fig. 3 in the main text, which can be attributed to their relatively high bulk modulus, as highlighted in Fig. S3. This effect is particularly pronounced in ZIF(nIM), where thermal conductivity and bulk modulus are notably high.

Additionally, the bonded interaction contributions of these two net topologies to thermal conductivity are significantly high, as shown in Fig. S5. Similar zigzag conduction pathways are observed in the z-direction of BCT (Fig. S6a) and in all three directions of SOD (Fig. S6b). This aligns with the exceptionally high thermal conductivity of BCT in the z-direction, while the isotropic SOD exhibits high thermal conductivity across all directions (Fig. S7b).

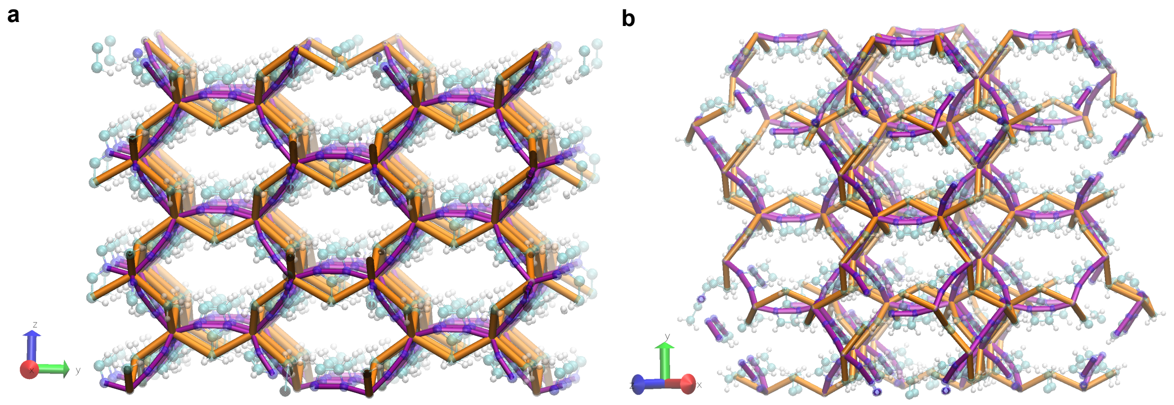

Supplementary Figure S6: Heat conduction pathways in ZIF(mIM) with net topology (a) BCT and (b) SOD. The primary and secondary pathways are represented by purple and orange tubes, respectively. Grey, blue, brown, and pink represent Zn, N, C, and H atoms, respectively. A secondary pathway is formed when atomic distances are shorter than 1.2 times the vdW radius[2], as visualized using the Visual Molecular Dynamics (VMD) package [7].

7 Thermal circuit conductivity fitting

As mentioned in the main text, the circuit conductivity is computed as

$$\sigma_i = \frac{J_i}{E_i} = \frac{I}{A_{\perp i}} \cdot \frac{L_i}{U}, \quad i = x, y, z, \tag{S3}$$

where σ_i is the electrical conductivity along direction i, J_i is the current density, E_i is the electric field, I is the current through the circuit, $A_{\perp i}$ is the cross-sectional area perpendicular to direction i of the lattice, L_i is the lattice length along i, and U is the applied voltage.

In the equivalent electrical circuit model, we assign each resistor a resistance of 1, and apply a voltage of 1, irrespective of the linker type. These choices can be regarded as reduced units. The circuit model can be characterized by a unit of length $l = 1\,\text{Å}$, a unit of voltage $u = 1\,\text{V}$, and a unit of resistance $r = r_{\text{linker}}\Omega$. Based on these basic units, all other units naturally follow. For instance, the unit of current is u/r, and the unit of conductivity is $1/lr$. The reduced units are summarized in Table S1. In terms of these reduced units, the reduced electrical conductivity is expressed as

$$\sigma_i^* = \frac{I^*}{U^*} \cdot \frac{L_i^*}{A_{\perp i}^*}, \quad i = x, y, z. \tag{S4}$$

, where L_i^*, $A_{\perp i}^*$, I^*, and U^* are dimensionless lattice length, cross-section area, current, and voltage, respectively.

Quantity	Reduced Units		Real Units
Length	$l^* = 1$	$\leftrightarrow$	$l = 1\,\text{Å}$
Current	$I^* = 1$	$\leftrightarrow$	$I = u/r_{\text{linker}}\text{A}$
Voltage	$U^* = 1$	$\leftrightarrow$	$U = 1\,\text{V}$
Resistance	$r^* = 1$	$\leftrightarrow$	$r = r_{\text{linker}}\Omega$
Conductivity	$\sigma^* = 1$	$\leftrightarrow$	$\sigma = 1/lr_{\text{linker}}\Omega^{-1}\,\text{Å}^{-1}$

Supplementary Table S1: Summary of reduced and real units used in the circuit model.

The reduced thermal circuit conductivity τ^* is an analogy to the derived dimensionless electrical conductivity σ^*, and they share the same numerical value. Fig. S7a shows the correlation between each linker's reduced thermal circuit conductivity and molecular

dynamics (MD) thermal conductivity. To convert the reduced thermal circuit conductivity to real units, we employ linear regression for each linker using `scikit-learn` python library[8]:

$$\kappa_i = \tau_{\text{linker}} \cdot \tau_i^*, \quad i = x, y, z, \tag{S5}$$

where τ_{linker} represents the scaling factor that converts the model-predicted dimensionless conductivity to the thermal circuit conductivity in real units τ_i. This scaling factor τ_{linker} depends on the linker type and is determined by fitting a linear regression model in each direction with an intercept fixed at zero, whose values are listed in Table S2. Figure S7b shows the correlation between the fitted thermal circuit conductivity and MD thermal conductivity in each direction. The data align on a single line, exhibiting a high Pearson coefficient of r=0.89.

**Reduced Units**		**Real Units** (W K^{-1} m^{-1})			
		x	y	z	total
$\tau^*_{\text{IM}} = 1$	↔	$\tau^x_{\text{IM}} = 0.054$	$\tau^y_{\text{IM}} = 0.054$	$\tau^z_{\text{IM}} = 0.056$	$\tau_{\text{IM}} = 0.055$
$\tau^*_{\text{dcIM}} = 1$	↔	$\tau^x_{\text{dcIM}} = 0.033$	$\tau^y_{\text{dcIM}} = 0.033$	$\tau^z_{\text{dcIM}} = 0.033$	$\tau_{\text{dcIM}} = 0.033$
$\tau^*_{\text{mIM}} = 1$	↔	$\tau^x_{\text{mIM}} = 0.067$	$\tau^y_{\text{mIM}} = 0.067$	$\tau^z_{\text{mIM}} = 0.067$	$\tau_{\text{mIM}} = 0.067$
$\tau^*_{\text{nIM}} = 1$	↔	$\tau^x_{\text{nIM}} = 0.056$	$\tau^y_{\text{nIM}} = 0.056$	$\tau^z_{\text{nIM}} = 0.057$	$\tau_{\text{nIM}} = 0.056$

Supplementary Table S2: Scaling factors (τ_{linker}) fitted using linear regression models with the intercept set to 0. The directional scaling factors τ^i_{linker} $(i = x, y, z)$ are derived from the thermal conductivity and reduced thermal circuit conductivity along each respective direction. The total scaling factor is determined using the average thermal conductivity and reduced thermal circuit conductivity. The total scaling factor aligns with the values obtained for each individual direction.

In Figs. 3, S7a and S7b, we present the Pearson correlation coefficients calculated after excluding the two exceptional net topologies, BCT and SOD, which exhibit atypical thermal transport behavior. For comparison, Table S3 summarizes the Pearson correlation coefficients computed both including and excluding these two net topologies. Our analysis indicates that, given the large number of topologies studied, the fitted scaling factors remain relatively robust to these outliers, whereas the Pearson correlation coefficients are more sensitive.

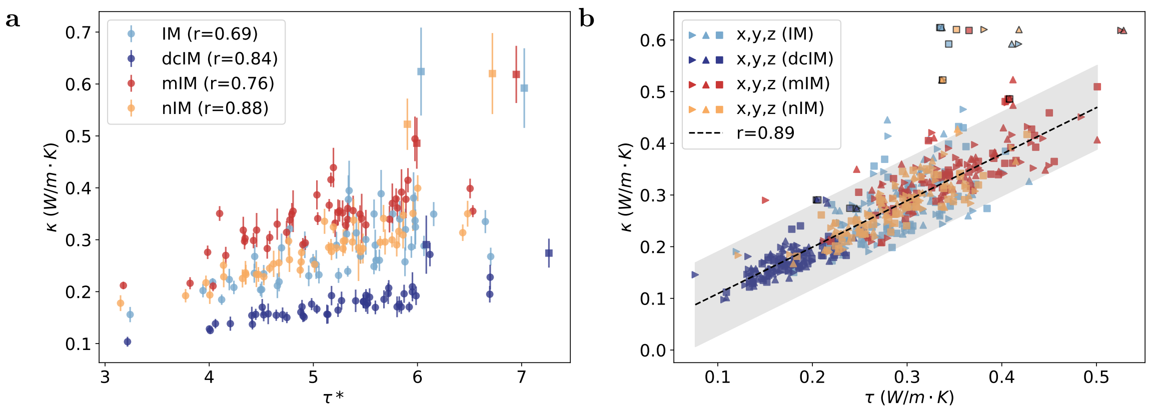

Supplementary Figure S7: The thermal conductivity as a function of (a) reduced thermal circuit conductivity and (b) fitted thermal circuit conductivity. The thermal circuit conductivity shows improved Pearson coefficients with thermal conductivity for each linker, except for ZIFs(mIM), than density. In (b), thermal conductivity in each direction is represented by different symbols. The fitted thermal circuit conductivity exhibits a strong correlation with MD thermal conductivity, with a Pearson coefficient of 0.89. All Pearson coefficients are computed excluding two exceptional net topologies, BCT and SOD, which are represented by squares in (b) and markers with black edges in (c).

Linker	Including	Excluding
IM	0.67	0.69
dcIM	0.83	0.84
mIM	0.79	0.76
nIM	0.78	0.88
Fitted τ	0.86	0.92

Supplementary Table S3: Comparison of Pearson correlation coefficients between thermal circuit predictions and MD thermal conductivity for different linkers, computed including and excluding the two exceptional net topologies, BCT and SOD.

8 Deviation of the thermal circuit prediction

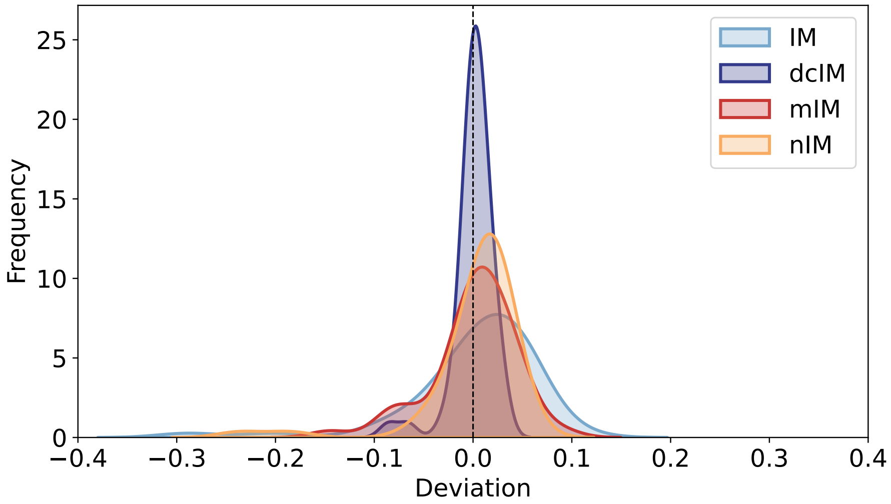

Supplementary Figure S8: The deviation distribution of the thermal circuit predictions. The deviation is defined as the difference between the thermal circuit conductivity and the MD thermal conductivity.

In Fig. S8, we present the deviation of the thermal circuit predictions, defined as the difference between the thermal circuit conductivity τ and the MD thermal conductivity κ. Overall, the deviation distributions exhibit an approximately normal shape with only a slight rightward shift, indicating that the thermal circuit model generally provides unbiased predictions. However, a few outliers, such as the MD thermal conductivity values of BCT and SOD, exert a disproportionate influence on the fitted scaling factors τ_{linker}. These outliers enlarge the fitted values of τ_{linker}, thereby shifting the predicted conductivities upward. This effect is most pronounced in IM, where the distribution shows a stronger positive bias, while in dcIM the influence of such exceptional values is more moderate, leading to only a minor shift.

9 Remarks on the circuit model

When mapping crystal networks to circuit models, the primary requirement is that the current remains independent of the unit cell choice. To achieve this, we break the periodic boundary condition (PBC) in the direction of conductivity calculation while maintaining PBC in other directions. By selecting atoms with bonds crossing the boundary as starting points and their translationally equivalent sites in the following adjacent cell as endpoints, we ensure that all bonds are accounted for and counted exactly once.

In addition, this approach of mapping crystal to circuit models can be extended to non-orthogonal cells with the following steps:

1. Since in the cell-vector basis, the unit cells are orthogonal, compute the current in the direction of the cell vectors as in the orthogonal case. This returns a current vector $\begin{pmatrix} I_1 \\ I_2 \\ I_3 \end{pmatrix}$, where I_i is the current in the direction of the $i-$th cell vector.
2. Use a basis transformation to obtain the current in $xyz-$direction, which is represented by $\begin{pmatrix} I_x \\ I_y \\ I_z \end{pmatrix}$:
   (a) Express the cell vectors in $xyz-$coordinates, obtaining the unit vectors $\hat{b}_1, \hat{b}_2, \hat{b}_3$;
   (b) Generate the 3×3 matrix $B = \left[\hat{b}_1; \hat{b}_2; \hat{b}_3\right]$, whose columns are the unit vectors $\hat{b}_1, \hat{b}_2, \hat{b}_3$;
   (c) The current in $xyz-$directions is $\begin{pmatrix} I_x \\ I_y \\ I_z \end{pmatrix} = B \cdot \begin{pmatrix} I_1 \\ I_2 \\ I_3 \end{pmatrix}$.
3. Use Eq. (S3) to compute the conductivity τ_x, τ_y, τ_z.

References

[1] Thomas F Willems, Chris H Rycroft, Michaeel Kazi, Juan C Meza, and Maciej Haranczyk. Algorithms and tools for high-throughput geometry-based analysis of crystalline porous materials. *Microporous and Mesoporous Materials*, 149(1):134–141, 2012.

[2] Seyed Mohamad Moosavi, Peter G Boyd, Lev Sarkisov, and Berend Smit. Improving the mechanical stability of metal–organic frameworks using chemical caryatids. *ACS central science*, 4(7):832–839, 2018.

[3] Paul Boone, Hasan Babaei, and Christopher E Wilmer. Heat flux for many-body interactions: corrections to lammps. *Journal of chemical theory and computation*, 15 (10):5579–5587, 2019.

[4] Matthew A Addicoat, Nina Vankova, Ismot Farjana Akter, and Thomas Heine. Extension of the universal force field to metal–organic frameworks. *Journal of chemical theory and computation*, 10(2):880–891, 2014.

[5] Damien E Coupry, Matthew A Addicoat, and Thomas Heine. Extension of the universal force field for metal–organic frameworks. *Journal of Chemical Theory and Computation*, 12(10):5215–5225, 2016.

[6] Donatas Surblys, Hiroki Matsubara, Gota Kikugawa, and Taku Ohara. Methodology and meaning of computing heat flux via atomic stress in systems with constraint dynamics. *Journal of Applied Physics*, 130(21), 2021.

[7] William Humphrey, Andrew Dalke, and Klaus Schulten. Vmd: visual molecular dynamics. *Journal of molecular graphics*, 14(1):33–38, 1996.

[8] F. Pedregosa, G. Varoquaux, A. Gramfort, V. Michel, B. Thirion, O. Grisel, M. Blondel, P. Prettenhofer, R. Weiss, V. Dubourg, J. Vanderplas, A. Passos, D. Cournapeau, M. Brucher, M. Perrot, and E. Duchesnay. Scikit-learn: Machine learning in Python. *Journal of Machine Learning Research*, 12:2825–2830, 2011.
